# YAP1 inhibition radiosensitizes triple negative breast cancer cells by targeting the DNA damage response and cell survival pathways

**DOI:** 10.18632/oncotarget.21913

**Published:** 2017-10-20

**Authors:** Daniel Andrade, Meghna Mehta, James Griffith, Janani Panneerselvam, Akhil Srivastava, Tae-Dong Kim, Ralf Janknecht, Terence Herman, Rajagopal Ramesh, Anupama Munshi

**Affiliations:** ^1^ Department of Radiation Oncology, The University of Oklahoma Health Sciences Center, Oklahoma City, Oklahoma 73104, USA; ^2^ Department of Pathology, The University of Oklahoma Health Sciences Center, Oklahoma City, Oklahoma 73104, USA; ^3^ Department of Cell Biology, The University of Oklahoma Health Sciences Center, Oklahoma City, Oklahoma 73104, USA; ^4^ Stephenson Cancer Center, The University of Oklahoma Health Sciences Center, Oklahoma City, Oklahoma 73104, USA

**Keywords:** yes associated protein 1, triple negative breast cancer, DNA damage, radiotherapy, verteporfin

## Abstract

The Hippo pathway is an evolutionarily conserved signaling pathway that regulates proliferation and apoptosis to control organ size during developmental growth. Yes-associated protein 1 (YAP1), the terminal effector of the Hippo pathway, is a transcriptional co-activator and a potent growth promoter that has emerged as a critical oncogene. Overexpression of YAP1 has been implicated in promoting resistance to chemo-, radiation and targeted therapy in various cancers. However, the role of YAP1 in radioresistance in triple-negative breast cancer (TNBC) is currently unknown. We evaluated the role of YAP1 in radioresistance in TNBC *in vitro*, using two approaches to inhibit YAP1: 1) genetic inhibition by YAP1 specific shRNA or siRNA, and 2) pharmacological inhibition by using the small molecule inhibitor, verteporfin that prevents YAP1 transcriptional activity. Our findings demonstrate that both genetic and pharmacological inhibition of YAP1 sensitizes TNBC cells to radiation by inhibiting the EGFR/PI3K/AKT signaling axis and causing an increased accumulation of DNA damage. Our results reveal that YAP1 activation exerts a protective role for TNBC cells in radiotherapy and represents a pharmacological target to enhance the anti-tumor effects of DNA damaging modalities in the treatment of TNBC.

## INTRODUCTION

Breast cancer is the most common cancer among women and the leading cause of cancer-related death worldwide. Despite advances in molecular classification and targeted molecular therapy, breast cancer incidence and mortality rate remain high [1–5]. TNBC is a subset of the basal-like breast cancer group, characterized by the lack of estrogen receptor (ER), progesterone receptor (PR) and Her-2/EGFR2 [2, 3]. TNBC is an invasive and aggressive breast cancer subtype that accounts for 10–15% of all breast cancers and is associated with poor prognosis, a high rate of recurrence, and distant metastasis. Because of the lack of approved targeted therapy, chemotherapy remains the mainstay of treatment for early and advanced disease. Therefore, better therapeutic tools and new treatment options for TNBC are urgently needed.

YAP1, an identified oncogenic transcriptional co-activator and a downstream mediator of the evolutionarily conserved Hippo pathway, has recently become a molecular target for cancer therapy. It is involved in the regulation of cell growth, proliferation, apoptosis, tumorigenesis, stem cell renewal and differentiation [6]. Central to the Hippo pathway is the highly conserved MST1/2-LATS1/2 kinase cascade. MST1/2, in complex with its coregulatory protein Salvador (SAV1), phosphorylates and activates LATS1/2. When the Hippo kinase cascade is activated, YAP1 is phosphorylated by LATS1/2 leading to cytosolic sequestration of YAP1 and subsequent proteasome-mediated degradation [7, 8]. When the Hippo kinase cascade is inactivated, YAP1 translocates to the nucleus where it binds to other transcription factors and promotes the expression of its target genes [7, 8].

The role of YAP1 in cancer remains controversial and conflicting reports on whether YAP1 functions as a tumor suppressor or as an oncogene have been reported in literature. Hyperactivation of YAP1 is wide-spread in cancers and YAP1 expression and nuclear localization strongly correlate with poor patient outcome and progression of cancer [9–13]. Further, aberrant activation of YAP1 signaling has been demonstrated to promote tumor growth, progression, and metastasis in many solid tumors [9–14]. YAP1 promotes oncogenesis by binding to the TEAD family of transcription factors and stimulating a downstream transcriptional program, leading to expression of anti-apoptotic and proliferation genes [15]. Although YAP1 behaves as an oncogene in several cancers, recent data suggest that YAP1 also has tumor suppressor functions in some contexts. Studies supporting YAP1 as a tumor suppressor include a) decreased YAP1 expression in several cancer cell lines and tumors; b) suppression of anoikis, increased cell migration and invasion and enhanced xenograft tumor growth and metastasis upon knockdown of YAP1; and c) induction of apoptosis in response to DNA damage by YAP1 in association with p73 [16–22].

While the different roles of YAP1 in oncogenesis might be tissue- and cell context-specific, elevated YAP1 signaling in cancer cells has been linked to anti-cancer therapy resistance to agents such as taxol, doxorubicin, cisplatin and tamoxifen [23–28]. Additionally YAP1 has been shown to contribute to resistance towards RAF- and MEK-targeted therapies and YAP1 depletion in cells harboring BRAF-V600E mutation sensitizes to RAF and MEK inhibitors [29]. Furthermore, YAP1 overexpression has been shown to promote radiation resistance in medulloblastoma and endometrial cancer cells [30, 31]. Based on these reports showing YAP1 role in cancer drug resistance, we hypothesized that YAP1 likely contributes to radiation therapy resistance in TNBC. The role of YAP1 in mediating radiation resistance in TNBC has thus far not been elucidated and makes it an attractive target for modulating radiosensitivity.

The results from our study offer direct evidence that YAP1 can function as a modulator of radiation resistance by suppressing accumulation of DNA damage and maintaining the EGFR and PI3K/AKT survival signaling, a pathway axis that has been shown to play crucial roles in resistance to therapy in TNBC.

## RESULTS

### YAP1 is overexpressed in TNBC cell lines and is predominantly nuclear

**Figure 1:**
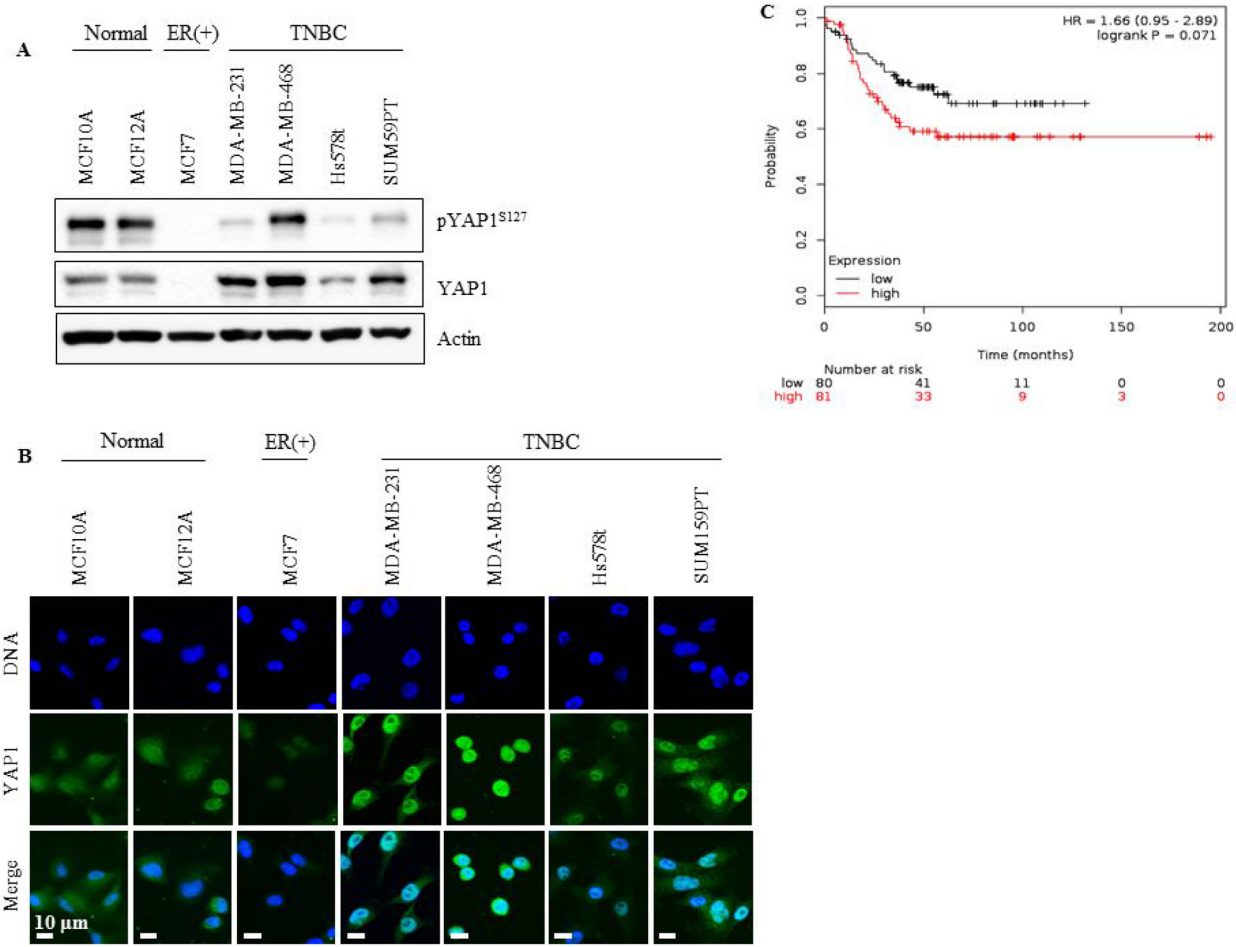
YAP1 is overexpressed in TNBC cell lines and correlates with low probability of relapse-free survival in TNBC patients (**A**) YAP1 and pYAP^S127^ status in TNBC cell lines (MDA-MB-231, MDA-MB-468, SUM159PT and Hs578t), normal cell lines (MCF-10a and MCF-12a) and an ER (+) cell line (MCF-7). (**B**) YAP1 localization assessed with immunofluorescent microscopy. (**C**) Kaplan Meier survival analysis of YAP1 mRNA expression level and the probability of relapse-free survival in TNBC patients.

We analyzed total and phosphorylated (p) YAP1 protein expression in a panel of human breast cancer cell lines that included ER (+), TNBC and normal cells. Abundant expression of total YAP1 was seen in TNBC cells compared with the other lines, as indicated by western blotting and immunofluorescent staining (Figure 1A and 1B). Phosphorylation of YAP1 at Ser127 (pYAP1^Ser127^) by LATS is known to promote its nuclear exclusion and cytoplasmic accumulation, leading to YAP inactivation. Reduced levels of pYAP1^Ser127^ were seen in TNBC cells suggesting a predominant nuclear localization of YAP1 indicative of activated YAP (Figure 1A), an observation confirmed by immunofluorescence analysis (Figure 1B).

### YAP1 expression is associated with relapse-free survival in TNBC patients

To evaluate the clinical relevance of YAP1 in TNBC, the effect of YAP1 mRNA expression on Relapse-Free Survival (RFS) was assessed. We performed Kaplan-Meier survival analysis using the on-line tool, Kaplan Meier plotter, which includes gene expression and survival data of about 4000 patients with breast cancer [32]. YAP1 mRNA expression was not significantly associated with RFS in patients with high or low expression of YAP1 (logrank *p* = 0.34, data not shown). However, when the analysis was restricted to TNBC patients, YAP1 mRNA expression correlated with decreased RFS (logrank *p* = 0.071, Figure 1C), supporting its role as an oncogene in TNBC.

### YAP1 inhibition reduces cell proliferation and impairs migration

**Figure 2:**
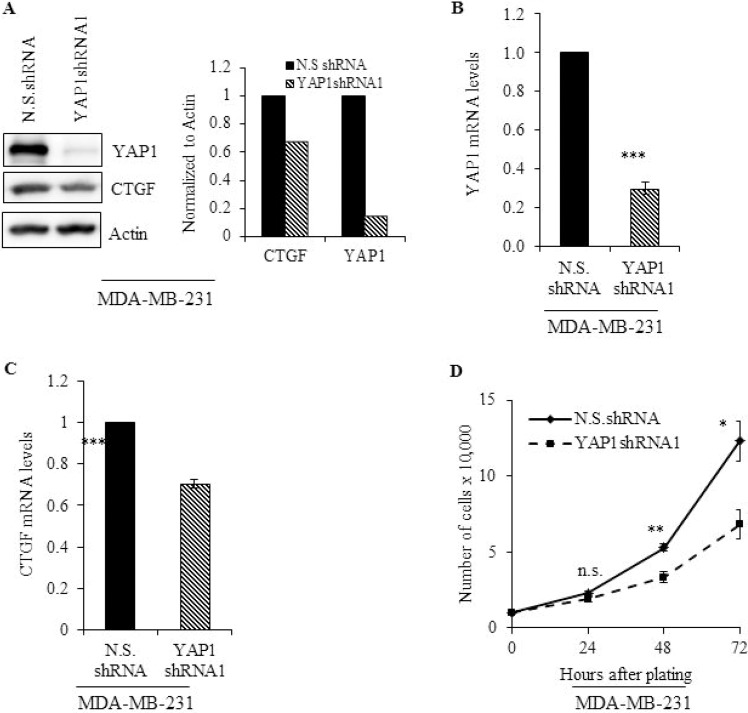
Genetic inhibition of YAP1 impairs cell proliferation MDA-MB-231 cells stably expressing a short hairpin RNA against YAP1 (YAP1shRNA1) were subjected to (**A**) immunoblot graph shows the intensity of the bands normalized to the N.S.shRNA lane] and (**B**-**C**) qRT-PCR analysis to evaluate protein and mRNA levels of YAP1 and its molecular target, CTGF. (**D**) Cell proliferation in N.S.shRNA and YAP1shRNA cells was evaluated at the indicated time points. Values shown are the means + SE (standard error) of three independent experiments. ^*^p ≤ 0.05, ^**^p ≤ 0.001, ^***^p ≤ 0.0001, n.s. = not significant.

MDA-MB-231 cells stably expressing a short hairpin (sh) RNA against YAP1 (YAP1shRNA1) were used to address the role of YAP1 in cell growth of TNBC. YAP1 protein and mRNA expression was greatly reduced in YAP1shRNA1 cells compared with vector control cells (N.S.shRNA) (Figure 2A and 2B). Furthermore, YAP1 downregulation reduced the expression of CTGF, a well-characterized YAP-targeted gene, at the protein and mRNA level (Figure 2A and 2C). The impact of YAP1 silencing on cell proliferation was also assessed. As shown in Figure 2D, YAP1 knockdown significantly reduced cell proliferation compared with the N.S.shRNA cells at 48 (*p* ≤ 0.0001) and 72 hours (*p* ≤ 0.05).

**Figure 3:**
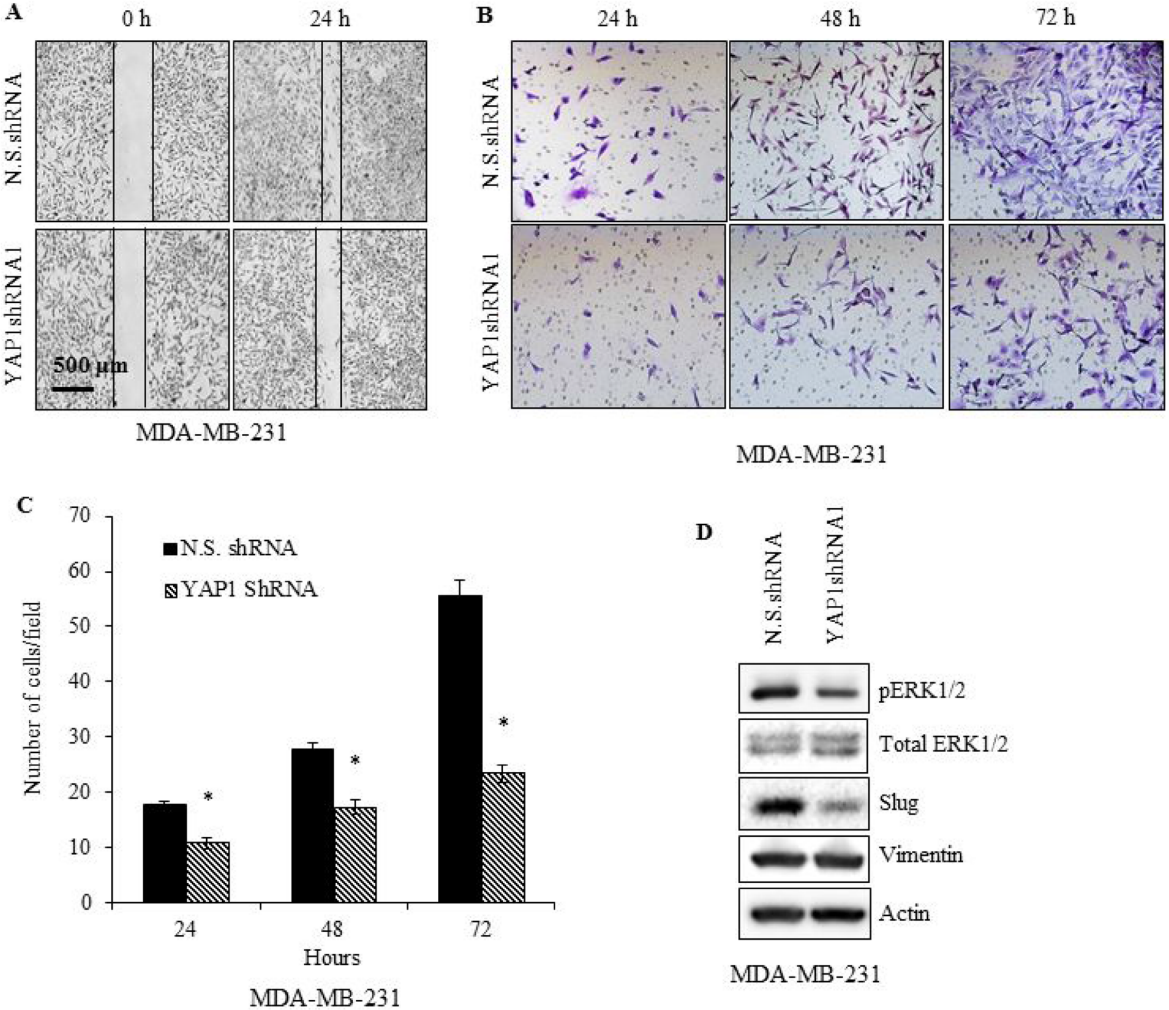
YAP1 silencing impairs MDA-MB-231 cell migration YAP1shRNA1 or N.S.shRNA cells were (**A**) evaluated at 0, and 24 h, for wound healing (**B**, **C**) *in vitro* migration ability via Matrigel-based transwell assay, and (**D**) immunoblot analysis of vimentin, Slug, and ERK. Data represent the average of three independent experiments. Error bars represent SEM (standard error of the mean). ^*^p ≤ 0.05.

We also determined the influence of YAP1 inhibition on MDA-MB-231 cell migration by performing wound healing and transwell migration assays. YAP1 knockdown significantly (*p* ≤ 0.05) impaired the wound healing capacity, as well as transwell migration (Figure 3A–3C) in MDA-MB-231 cells. A decrease in migration could be a reflection of reversion from mesenchymal state to epithelial state following YAP1 downregulation. YAP1 downregulation resulted in the conversion of cells from a mesenchymal to an epithelial-like morphology with a cobblestone-like appearance, suggesting a potential reversion to an epithelial state (data not shown). Expression of Slug and ERK, critical regulators of cell migration and invasion in TNBC cells showed a marked decrease upon YAP1 downregulation (Figure 3D) [33, 34]. Although no obvious difference in vimentin levels was detected, reduction in the expression levels of pERK1/2 and Slug could partly explain the impaired migration upon YAP1 downregulation. However, while Slug expression is crucial for the repression of E-cadherin, we did not observe any recovery in the expression of E-cadherin following YAP1 downregulation (data not shown) [35]. This could be because the E-cadherin promoter is hypermethylated in MDA-MB-231 cells, and de-repression of the E-cadherin promoter could require participation of factors not regulated by YAP1 [36]. Altogether our results show that YAP1 inhibition in TNBC cells results in reduced cell proliferation and migration with potential transition from a mesenchymal to an epithelial state.

### Inhibition of YAP1 radiosensitizes TNBC cells

**Figure 4:**
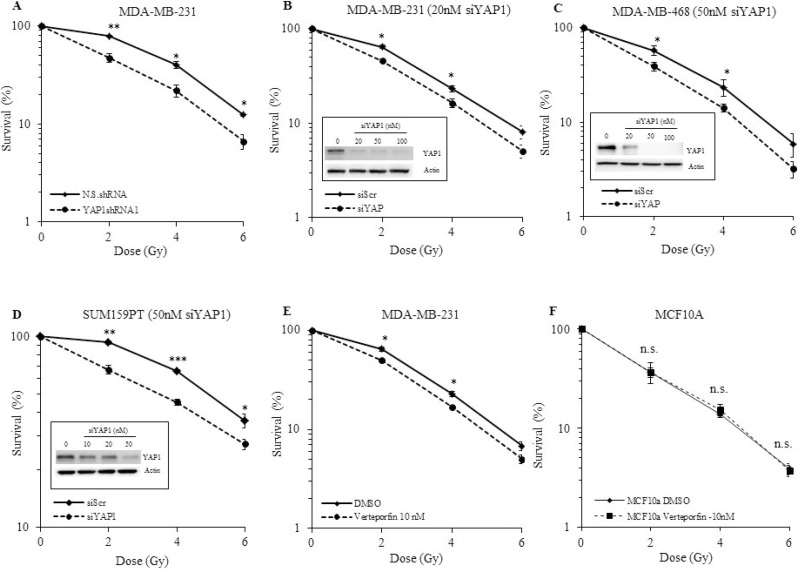
Effect of YAP1 inhibition on radiosensitivity of TNBC and normal cell lines (**A**) YAP1shRNA1 cells show a significant reduction in the surviving fraction compared with the N.S.shRNA controls. (**B**-**D**) Transient inhibition of YAP1 by a pool of siRNAs sensitizes MDA-MB-231, MDA-MB-468 and SUM159PT to radiation. (**E**) Pharmacological inhibition of YAP1 by verteporfin radiosensitizes MDA-MB-231 cells while having no effect on MCF-10A. (**F**) Values shown are the means + SE of three independent experiments. ^*^p ≤ 0.05, ^**^p ≤ 0.001, ^***^p ≤ 0.0001, n.s. = not significant.

Studies have shown that YAP1 plays a role in radioresistance [30, 31]. We investigated the effect of YAP1 silencing using shRNA and siRNA on the radiosensitivity of TNBC cell lines (MDA-MB-231, MDA-MB-468, and SUM159PT) by assessing their clonogenic potential. MDA-MB-231-YAP1shRNA1 cells were significantly more sensitive to the cytotoxic effects of radiation than N.S.shRNA cells (Figure 4A, *p* ≤ 0.05). The degree of radiosensitization was quantified from the survival curves by comparing the surviving fractions at the radiation dose of 2 Gy (SF2) and by calculating the dose enhancement factor (DEF), i.e. the ratio of radiation doses to achieve a given survival level. Significant differences in survival between YAP1shRNA and N.S.shRNA were observed at all three doses of radiation (Figure 4A, *p* ≤ 0.05). Furthermore, two other independent YAP1shRNAs also significantly sensitized MDA-MB-231 cells to radiation exposure (Supplementary Figure 1). To further test the effect of YAP1 genetic inhibition on radiosensitization and to discard any potential molecular re-wiring due to stable inhibition of YAP1, we used a pool of three target-specific siRNAs against YAP1 (siYAP1) and compared them with non-targeted siRNA (siScr). Consistent with YAP1shRNA results, siRNA-mediated inhibition of YAP1 significantly radiosensitized all three TNBC (MDA-MB-231, MDA-MB-468, and SUM159PT) cell lines tested (Figure 4B–4D, *p* ≤ 0.05). To further examine whether pharmacologic inhibition of YAP1 generates a similar radiosensitizing effect we used verteporfin, a small molecule inhibitor of YAP1. Verteporfin radiosensitized the MDA-MB-231 cells but had no effect on the normal human mammary epithelial cell line, MCF10A, further highlighting the relevance of YAP1 expression in radioresistance of TNBCs (Figure 4E and 4F). The DEF values for all the cell lines tested are provided in Table 1. These results indicate that genetic and pharmacological inhibition of YAP1 radiosensitizes the TNBC cell lines examined, suggesting that YAP1 is a promising molecular target for radiation sensitization of TNBC.

**Table 1:** Dose enhancement factor (DEF) values calculated from the survival curves shown in Figure 4

Cell Line	Treatment	DEF
MDA-MB-231	YAP1shRNA1	1.22
MDA-MB-231	siYAP (10 nM)	1.18
MDA-MB-468	siYAP (50 nM)	1.17
SUM159PT	siYAP (50 nM)	1.33
MDA-MB-231	Verteporfin (10 nM)	1.18
MCF10A	Verteporfin (10 nM)	1.00

DEF were calculated by dividing the radiation dose that produced 10% cell survival in control cells by that of the treated cells.

### Radiation promotes YAP1 nuclear translocation

**Figure 5:**
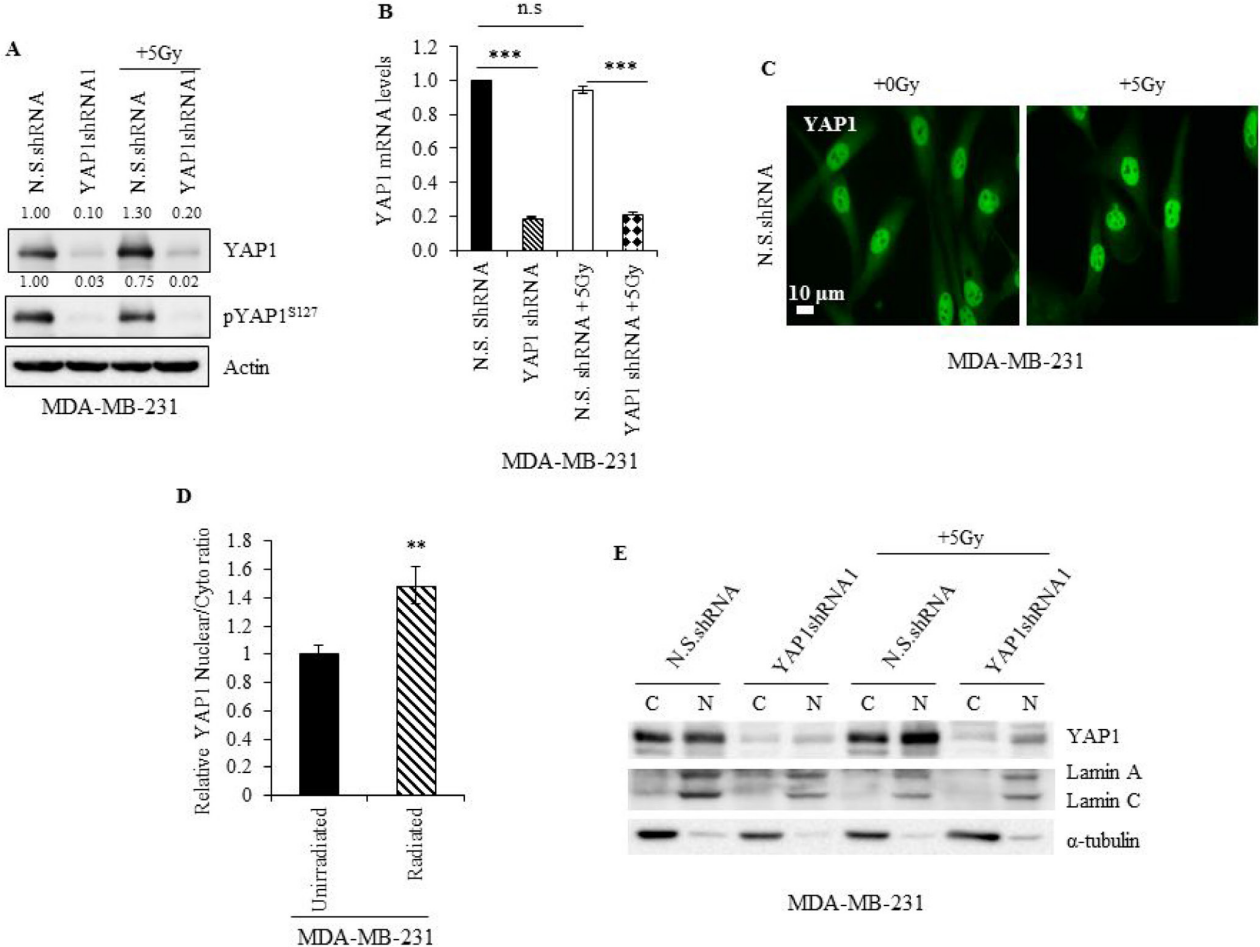
Radiation promotes YAP1 stabilization and nuclear translocation in TNBC cells (**A**) Immunoblot (**B**) qRT-PCR and (**C**) immunofluorescence analysis of YAP1 in lysates of non-irradiated and irradiated N.S.shRNA and YAP1shRNA1 cells. Numbers represent the intensity of the bands normalized to the N.S. shRNA lane. (**D**) Ratio of nuclear and cytoplasmic YAP1 intensities measured from immunofluorescence microscopy images as shown in (C). (**E**) Immunoblot analysis of YAP1 in cytoplasmic (C) and nuclear (N) fractions of non-irradiated and irradiated N.S.shRNA and YAP1shRNA1 cells. Values shown are the means + SE of three independent experiments. ^**^p ≤ 0.001 and ^***^p ≤ 0.0001.

**Figure 6:**
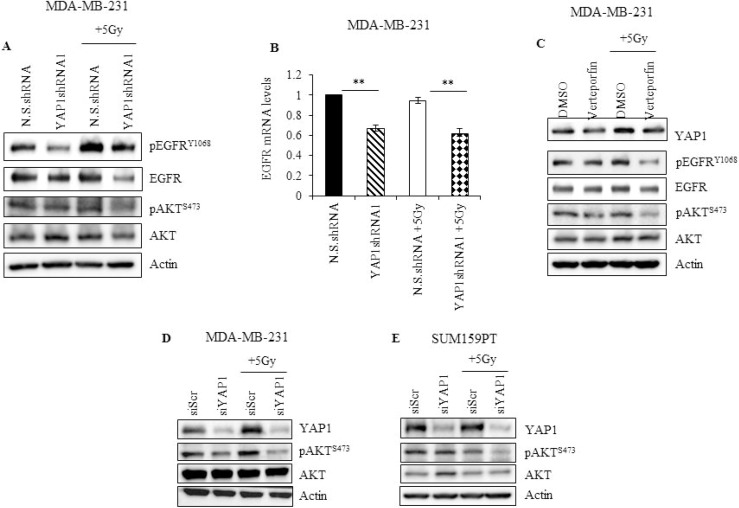
YAP1 activity is required to sustain EGFR and PI3K/AKT signaling during radiation in TNBC cells (**A**) YAP1 shRNA or N.S. shRNA cells were irradiated and cell lysates, obtained two hours after radiation, and analyzed for pEGFR^Y1068^, total EGFR, pAKT^S473^, and total AKT (**B**) mRNA obtained from cells treated as described in (A) was analyzed for EGFR expression by qRT-PCR (**C**) MDA-MB-231 cells treated with 10 nM of verteporfin for 24 hours and irradiated. Lysates were prepared two hours later, and analyzed for YAP1, pEGFR^Y1068^, total EGFR, pAKT^S473^, and total AKT. (**D**, **E**) MDA-MB-231 and SUM159PT cells treated with YAP1 siRNA for 24 hours were subjected to radiation. Cells were collected 2 hours later and lysates were analyzed for YAP1, pAKT^S473^, and total AKT. Actin was used as loading control. Values shown are the means + SE of three independent experiments. ^**^p ≤ 0.001.

**Figure 7:**
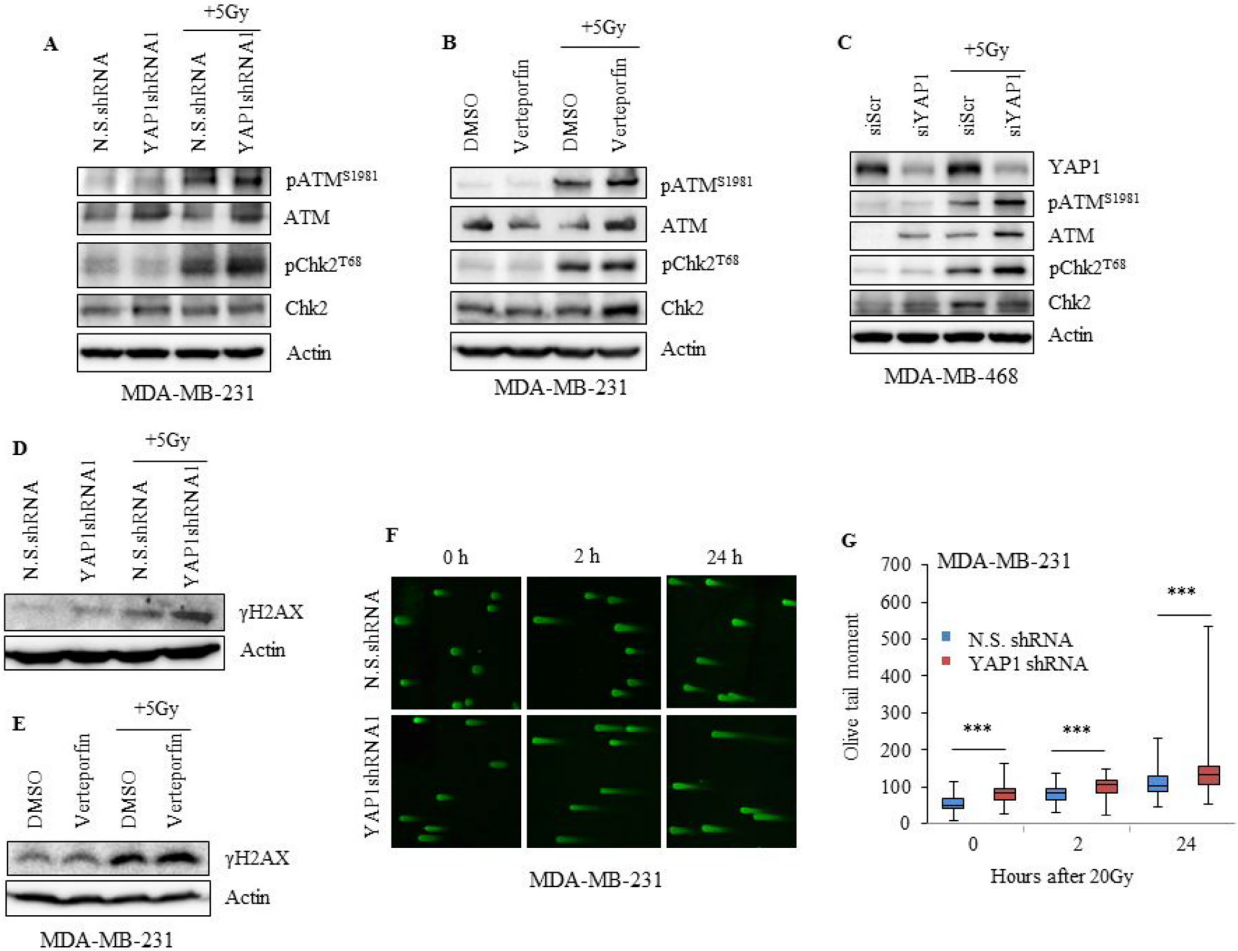
Inhibition of YAP1 impairs the DNA damage response in TNBC cells (**A**) MDA-MB-231 YAP1shRNA1 or N.S.shRNA cells (**B**) verteporfin-treated (10 nM 24 h) MDA-MB-231 and (**C**) YAP1 siRNA treated MDA-MB-468 cells were irradiated, and lysates were analyzed for pATM^S1981^, total ATM, pChk2^T68^, and total Chk2. (**D**, **E**) Lysates of cells treated as in (A) and (B) were evaluated for γH2AX. (**F**, **G**) DNA damage was assessed by neutral comet assay and tail moment was quantified. Actin served as loading control. Values shown are the means + SE of three independent experiments. ^***^p ≤ 0.0001.

To investigate the effect of radiation on YAP1, we evaluated YAP1 expression in irradiated N.S.shRNA MDA-MB-231 cells and compared it to the non-irradiated cells. An increase in YAP1 protein was observed in irradiated N.S.shRNA cells (Figure 5A) and in irradiated siScr- MDA-MB-231 and MDA-MB-468 cells (Figure 6D and 7C) compared with non-irradiated cells. No significant difference in YAP1 mRNA levels was observed between irradiated and unirradiated N.S.shRNA and YAP1shRNA1 cells (Figure 5B), suggesting that the observed increase in YAP1 protein is likely due to post-translational regulation. Accordingly, a reduction in the pYAP1^Ser127^ was seen in irradiated N.S.shRNA MDA-MB-231 cells (Figure 5A) compared to non-irradiated cells. YAP1 mRNA and protein expression in MDA-MB-231 YAP1shRNA cells remained unaffected when subjected to radiation and compared to non-irradiated cells (Figure 5A and 5B).

Phosphorylation of YAP1 at Ser127 is known to prevent its translocation from the cytoplasm to the nucleus and prime it for proteosomal degradation [7, 8]. Since in our study we observed a reduction in pYAP1^S127^ protein levels in irradiated N.S.shRNA MDA-MB-231 cells, it is likely that radiation promotes increased YAP1 nuclear translocation. Immunofluorescence studies showed irradiated N.S.shRNA MDA-MB-231 cells displayed higher YAP1 nuclear-to-cytoplasmic ratio than non-irradiated cells, indicating that YAP1 accumulated in the nuclei of irradiated cells (Figure 5C and 5D; *p* ≤ 0.001). This observation was further validated by fractionation studies, which showed increased YAP1 protein levels in the nucleus of N.S.shRNA MDA-MB-231 irradiated cells compared to non-irradiated cells (Figure 5E). An increase in nuclear YAP1 protein was also observed in YAP1shRNA1 irradiated cells compared to non-irradiated cells. Though irradiated siScr-SUM159PT cells showed no appreciable increase in YAP1 levels compared to non-irradiated siScr-SUM159PT cells by western blotting (Figure 6E), an increased nuclear accumulation of YAP1 upon radiation was observed by immunofluorescence and fractionation studies (Figure 6E and Supplementary Figure 2A–2C). Collectively, our results indicate that radiation promotes YAP1 activation and its translocation to the nucleus in TNBC cells.

### YAP1 activity is required to sustain survival signaling upon radiation

To further understand the mechanism by which YAP1 protects TNBC cells from radiation-induced cytotoxicity, we assessed the involvement of other survival factors associated with YAP1. In esophageal cancer, YAP1 induces EGFR expression by binding to the TEAD binding site in the EGFR promoter [23]. In cervical cancer and hepatocellular carcinoma, YAP1 can activate EGFR signaling by upregulating the expression of TGF-α or AREG [37, 38]. To determine whether there is an interaction between YAP1 and EGFR signaling in irradiated TNBC cells, protein levels of EGFR and its activated phosphorylated form were evaluated. Genetic (YAP1shRNA1) and pharmacological (verteporfin) inhibition of YAP1 led to downregulation of pEGFR^Y1068^ and total EGFR at both protein and mRNA level in MDA-MB-231 cells (Figure 6A–6C). Furthermore, radiation-induced activation of EGFR was greatly reduced in YAP1shRNA1 or verteporfin-treated MDA-MB-231 cells compared to N.S.shRNA and DMSO-treated cells respectively. Additionally, overexpression of YAP1 in MDA-MB-231 cells led to a marked increase in both pEGFR^Y1068^ and total EGFR expression (Supplementary Figure 3). These results demonstrate a cross-talk between YAP1 and EGFR signaling, and suggest that activated YAP1 could protect TNBC cells from radiation, in part, by promoting EGFR-mediated cell survival signaling, which can be suppressed by genetic or pharmacological inhibition of YAP1 (Figure 6A and 6C).

Next we examined for AKT expression, a known downstream target of EGFR signaling. YAP1 is known to activate PI3K by transcriptional regulation of Pik3cb, the downstream target of which is AKT [39]. As shown in Figure 6A, and 6C–6E, there was no obvious change in pAKT^Ser473^ levels in N.S.shRNA, DMSO-treated and siScr-treated TNBC cells with and without exposure to radiation. YAP1 knockdown alone using shRNA, verteporfin or siRNA also did not inhibit pAKT^Ser473^ levels (Figure 6A, 6C–6E). However, YAP1 inhibition using shRNA, siRNA, or verteporfin in combination with radiation caused a dramatic decrease in pAKT^Ser473^ levels (Figure 6A, 6C–6E). Finally, overexpression of YAP1 markedly increased pAKT^Ser473^ in MDA-MB-231 cells (Supplementary Figure 3). These results suggest that both EGFR and PI3K signaling converge on AKT and that AKT signaling heavily depends on YAP1 activity upon radiation.

Together, our results demonstrate that radiation enhances nuclear translocation of YAP1. Further, radiation-induced activation of YAP1 augments EGFR and PI3K/AKT-mediated survival signaling, which can be suppressed by genetic or pharmacological inhibition of YAP1, further lending support for development of YAP1 as a therapeutic target for radiosensitization of TNBC.

### YAP1 inactivation impairs the DNA damage response

To further understand the molecular basis by which YAP1 inhibition renders TNBC cells sensitive to radiation, we evaluated the involvement of DNA damage response (DDR) in MDA-MB-231 and MDA-MB-468 cells. Knockdown of YAP1 in combination with radiation led to an increase in pATM^S1981^ and its effector target pChk2^T68^ in both cell lines, an event reflecting the activation of ATM by DNA damage (Figure 7A–7C) [40]. Since γ-H2AX is also an indicator of radiation-induced DNA-double stranded breaks (DSBs), we evaluated the overall effect of YAP1 inhibition on radiation-induced γ-H2AX by western blot analysis. Compared to N.S.shRNA, a significant increase in γ-H2AX levels was seen in YAP1shRNA1 cells following radiation (Figure 7D). Increase in γ-H2AX levels was also observed in MDA-MB-231 cells when treated with verteporfin in combination with radiation, further supporting the effect of YAP1 inhibition on DDR (Figure 7E). As an additional measure of the effects of YAP1 inhibition on radiation-induced DSBs, we performed a neutral comet assay in MDA-MB-231 cells. Our results showed that inhibition of YAP1 led to significant increase in DNA damage compared with N.S.shRNA control (Figures 7F-7G; *p* ≤ 0.0001). However, maximum DNA damage was observed at 24 h after radiation treatment in YAP1shRNA cells compared to N.S.shRNA cells (Figure 7F).These results demonstrate that YAP1 inhibition suppresses the repair of radiation-induced DSBs.

## DISCUSSION

Despite the efforts to improve treatments against the most aggressive form of invasive breast cancer, TNBC patients continue to exhibit poor survival with half of them developing resistance to therapy. YAP1 is a well characterized transcriptional co-activator and is one of the two main downstream effectors of the Hippo pathway [7, 8]. YAP1 expression is elevated in a number of human malignancies and its expression has been suggested as a negative prognostic factor for cancer [10–14]. A recent study of patients with TNBC showed that YAP1 expression in tumor cells and the surrounding stroma is associated with a decreased likelihood to achieve pathological complete response (pCR) [41]. The findings also illustrated that YAP1 expression was associated with higher tumor grade in patients with TNBC, further highlighting the role and the relevance of YAP1 in the pathology of TNBC [41]. We analyzed a publically available dataset for YAP1 expression and established that YAP1 expression had a negative effect on relapse-free survival (RFS) in TNBC patients [32]. It is however to be noted that the type of treatments including radiation therapy received by these TNBC patients are not known and hence it cannot be assumed that all patients received radiation treatment. While this could be viewed as a caveat in the study nevertheless our results provide evidence that a correlation between YAP1 and RFS in TNBC exists.

In this study we demonstrate that YAP1 plays an important role as an oncogene in the pathology of TNBC. Compared with ER positive breast cancer cells and normal cells, YAP1 expression was predominantly observed in the nucleus and at higher levels in TNBC cells. Increasing evidence suggests that elevated YAP1 expression correlates with epithelial-mesenchymal transition (EMT) marker expression, whereas suppression of YAP1 decreases EMT marker expression and impedes tumor migration and invasion, suggesting a critical role for YAP1 in promoting metastasis and cancer stemness. We found that YAP1 silencing led to impairment of cellular proliferation and tumor cell migration. Moreover, silencing YAP1 was associated with changes in cell morphology and decreased levels of mesenchymal markers including Slug and ERK1/2 which are critical regulators of cell migration and invasion in TNBC cells [33, 34].

Various studies have reported that YAP1 overexpression mediates resistance to chemotherapeutic agents (such as cisplatin, doxorubicin, paclitaxel and MAPK pathway inhibitors), EGFR inhibitors and radiation therapy [23–27, 30, 31]. Subsequently, several studies have reported that a) high levels of YAP expression predict poor response to radiation therapy; b) YAP knockdown potentiates DNA damage response and increases sensitivity to radiation treatment; and c) YAP activation induces resistance to radiation [28, 30, 31, 42]. In accordance with these studies, we found that silencing YAP1 using genetic approaches abrogated the resistance of TNBC cells to radiation, highlighting the importance of YAP1 as a potential target for radiation therapy in TNBC patients. Three different short hairpin RNAs against YAP1 were tested to rule out any off-target effects. Further, to exclude any potential effect of signaling re-wiring due to stable expression of YAP1shRNAs, transient knockdown of YAP1 was achieved using YAP1siRNA. Several studies have reported that verteporfin can inhibit YAP1 transcriptional activity thereby leading to tumor growth suppression and sensitization to cytotoxic agents in a variety of tumor types, such as esophageal cancer, rhabdomyosarcoma, ovarian cancer and bladder cancer [23, 28, 43, 44]. Our data also indicate that verteporfin restored radiation sensitivity in TNBC cells. Interestingly, MCF10A cells that express modest levels of YAP1 when subjected to verteporfin treatment did not exhibit sensitivity to radiation. One possible explanation for the lack of radiosensitization is due to the inefficient nuclear translocation of YAP1 in MCF10A. This interesting possibility has not been tested in the present study and will need to be investigated in future studies. Nevertheless our study results show that the cytotoxic effect of verteporfin is restricted to cancer cells, a feature that is preferred in cancer treatment.

Genetic insults, such as radiation can cause a series of DNA lesions among which DNA double-strand breaks (DSBs) are the most critical and lethal. In the presence of DSBs, ATM, a DSB sensor, gets activated through autophosphorylation of the Ser1981 residue and activates the distal transducer kinase, Chk2 [45]. Ectopic expression of YAP1 has been shown to promote rapid dephosphorylation of ATM and Chk2, allowing the cells to eventually override the G2/M checkpoint and enter mitosis in the presence of damaged DNA [30]. Our results also indicate that YAP1 inhibition causes sustained DNA damage response signaling, as evidenced by activation of ATM and Chk2 after radiation. Further, comet assay revealed that YAP1-silenced cells had larger and longer-lasting tails than control cells, indicating higher levels of DNA damage. Next, we found that YAP1 silencing led to upregulation of γ-H2AX indicating that YAP1 inhibition suppressed DNA repair after irradiation. Our results indicate that YAP1 inhibition in TNBC cells elicits DNA damage and inhibits DNA repair resulting in radiosensitization thereby revealing the potential of targeting YAP1 during DNA damage-inducing therapies in TNBC.

Radiation-induced activation of YAP1 was found to correlate with activation of EGFR and PI3K signaling. Our results concur with previous reports that YAP1 promotes the activation of EGFR and PI3K/AKT signaling [23, 37–39]. While neither activation nor inhibition of YAP1 seem to have an obvious effect on the pAKT levels, a combined treatment of radiation and YAP1 inhibition caused a dramatic inhibition of pAKT, indicating that AKT signaling is highly dependent on YAP1 to sustain its activity during radiation. These findings are of great importance for TNBC therapy as several studies, in an effort to identify promising target molecules, have found the EGFR and PI3K/AKT/mTOR signaling pathways to be predominantly altered in TNBC patients [46–49]. Although inhibitors of the PI3K/AKT, mTOR and EGFR are currently available, they are not very efficient or present great concerns in the treatment of TNBC patients, especially increased toxicity, that have led to trial suspensions in some cases [48–50]. Our results show that genetic or pharmacological inhibition of YAP1 is a promising new way to target these critical pathways.

In summary, our study provides evidence that YAP1 inhibition modulates cell survival signaling pathways and sensitizes TNBC cells to radiation therapy. Our work provides basis for advanced testing of YAP1 inhibition in radioresistant TNBCs and determining its efficacy *in vivo*.

## MATERIALS AND METHODS

### Cell lines

MDA-MB-231 and MDA-MB-468 cells were maintained in alpha-MEM (CellGro, Manassas, VA) containing 10% fetal bovine serum (FBS), L-glutamine (2 mmol/L), and penicillin-streptomycin (2 mmol/L). SUM159PT cells were maintained in Ham's F-12 media supplemented with 5% heat-inactivated FBS, penicillin-streptomycin (2 mmol/L), 10 mM Hepes, and 1μg/ml insulin. MCF-10A, MCF-12A, MCF-7 and Hs578t were maintained in media recommended by the supplier. All cultures were maintained at 37°C in an atmosphere of 5% CO2. SUM159PT cells were obtained from Asterand (Detroit, MI). All other cell lines were procured from the American Type Culture Collection (ATCC, Manassas, VA).

### Preparation of stable YAP1 knockdown cells

All shRNA constructs were cloned into pSIREN-RetroQ (Clontech). The preparation of these vectors and the procedures for production of retrovirus and infection of target cells with viral particles containing these shRNAs have been described previously [51]. The human sequences targeted by these shRNAs are as follows: YAP1 #1 (CCACCAAGCTAGATAAAGA); YAP1 #2 (GCTTATAAGGCATGAGACA); and YAP1 #3 (AGTAATAGTTGGTTGTGAA).

### RNA interference and verteporfin

For transient YAP1 inhibition, cells (1 × 10^5^ cells/ 35 mm dish) were transfected with YAP1 siRNA (Santa Cruz Biotechnology, Inc.) using DharmaFECT 1 Transfection Reagent (GE Healthcare Dharmacon Inc) according to the manufacturer's instructions. Pharmacological inhibition of YAP1 was performed by incubating cells with 10 nM verteporfin (Sigma-Aldrich) for 24 hours.

### Immunofluorescence staining

Cells grown on micro-cover glasses (5 × 10^4^ cells/coverglass/35 mm dish; VWR international) were irradiated at 5Gy. Two hours after radiation, they were fixed with 4% paraformaldehyde for 20 min and stained as previously described [52]. We used a rabbit anti-YAP1 antibody (dilution 1:100, Cell Signaling Technology) as a primary antibody and an Alexa Fluor^®^ 488 goat anti-rabbit antibody (dilution 1:200, Invitrogen) as a secondary antibody.

### Wound healing assay

Plated cells (MDA-MB-231 cells; 3 × 10^5^ cells/ 60 mm dish) were grown to 95% confluency and wounded longitudinally, using a 200-μl pipette tip. Twenty-four and forty-eight hours later, cells were stained with crystal violet and analyzed to determine any defects in cell migration. Experiments were performed in triplicate.

### Cell migration assay

MDA-MB-231 cells (5 × 10^3^ cells) were seeded in the upper chamber of the Transwell (8 μm; BD Biosciences, Bedford, MA) in medium containing 2% FBS and placed in a six-well plate filled with 2 ml of medium containing 20% FBS (lower chamber). After 24, 48, and 72 h incubation, the inserts were removed and stained with crystal violet. The number of migrated cells was counted and data shown is a representative of three independent experiments.

### Clonogenic survival

Colony-forming ability was assayed as described previously [53, 54]. Briefly, breast cancer (MDA-MB-231, MDA-MB-468, SUM159PT) and normal (MCF10A) cells (1 × 10^5^ cells/ 35 mm dish) were exposed to a single dose of radiation with the indicated doses and incubated for 10 days. Colonies were stained with crystal violet. Colonies consisting of more than 50 cells were counted. The percentage plating efficiency (PE) and fraction surviving a given treatment was calculated based on the survival of non-irradiated cells from each treatment.

### Western blot analysis

Protein extracts were separated by SDS-PAGE, transferred onto PVDF membranes and probed with polyclonal rabbit antibodies against YAP1, pYAP1^S127^, pEGFR^Y1068^, EGFR, pAKT^S473^, AKT, pERK, ERK, Slug, p73, Vimentin, CTGF, Lamin A/C, Tubulin, pATM^S1981^, ATM, pCHK2^T68^, CHK2, β-actin (Cell Signaling Technology) and γH2AX (EMD Millipore Corporation).

### Quantitative polymerase chain reaction

Total RNA was isolated using TRIzol (Life Technologies, Grand Island, NY) and subjected to reverse transcription using the Omniscript RT Kit (Qiagen Inc., CA). The resulting cDNA was used for quantitative PCR (Bio-Rad CFX96™ TouchReal-Time PCR Detection System) with PerfeCTa SYBR Green Fast Mix (Quanta Biosciences, MD). Cycle threshold (C_t_) numbers from the gene being evaluated and GAPDH were converted to relative gene expression values using the 2^-ΔΔCt^ method in triplicate experiments.

### Cell fractionation

MDA-MB-231 and SUM159PT cells (1 × 10^6^ cells) were seeded in 10-cm plates in triplicate. Two hours after radiation, nuclear and cytoplasmic fractions were prepared using the NE-PER™ Nuclear and Cytoplasmic Extraction Reagents kit (ThermoFisher Scientific) following the manufacturer's instructions.

### Neutral comet assay

DNA damage in MDA-MB-231 cells was assessed using a Comet Assay kit (Trevigen, Gaithersburg, MA) according to the manufacturer's instructions. Briefly, NS shRNA and YAP1shRNA cells were subjected to radiation and analyzed for DNA damage at 2 h and 24 h post radiation. The presence of comet tails was determined with a Nikon fluorescence microscope. The tail moment was calculated as: (percentage of the DNA in the tail) × (tail length), where the percentage of DNA in the tail and tail length were quantified with Casplab comet assay software.

### Statistical Analysis

Statistical analysis was performed using the *t*-test (Sigma Plot 5.02v) and described as mean ± standard error of the mean. A difference was regarded as significant if *p* < 0.05.

## SUPPLEMENTARY MATERIALS FIGURES


