# Supplementary material for: YAP1 inhibition radiosensitizes triple negative breast cancer cells by targeting the DNA damage response and cell survival pathways
Source: Oncotarget. 2017 Oct 20;8(58):98495–508. doi: 10.18632/oncotarget.21913 (PMC5716745; doi:10.18632/oncotarget.21913)
Supplement: Supplementary file 1 [file oncotarget-08-98495-s001.pdf]

YAP1 inhibition radiosensitizes triple negative breast cancer cells by targeting the DNA damage response and cell survival pathways

SUPPLEMENTARY MATERIALS

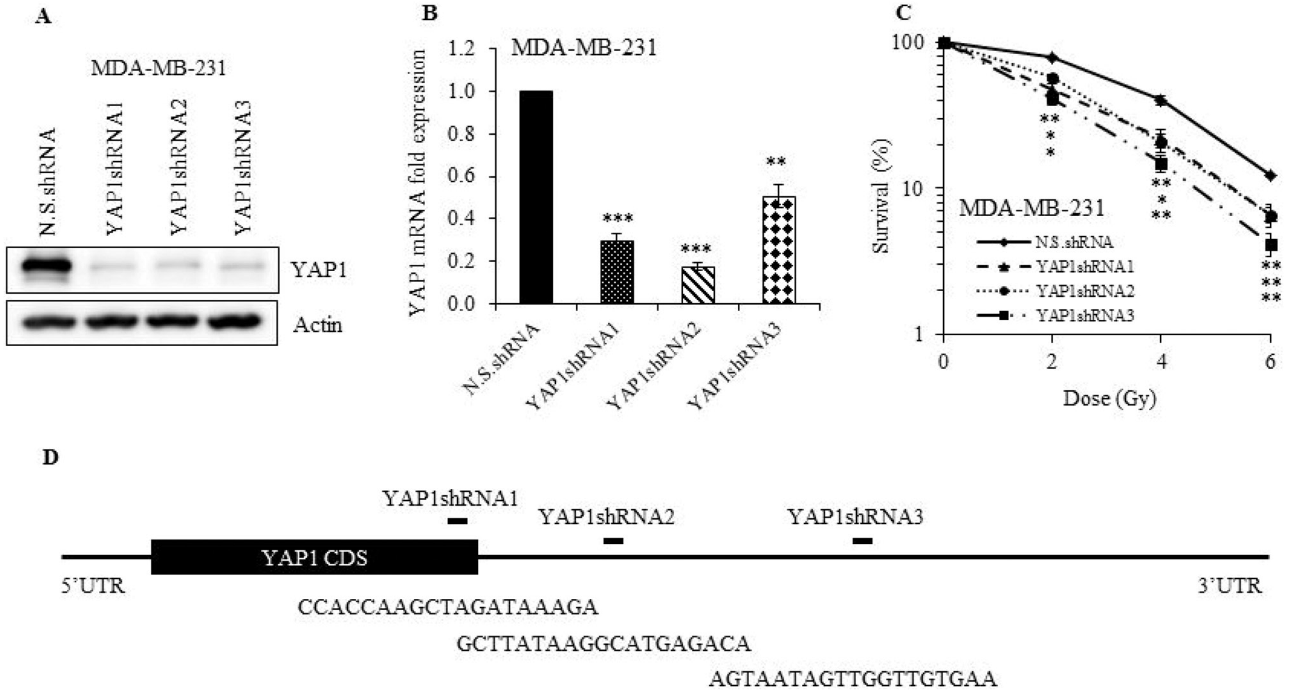

Supplementary Figure 1: Stable YAP1 inhibition sensitizes the MDA-MB-231 TNBC cell line to radiation. Downregulation of YAP1 in MDA-MB-231 cells with three different shRNAs was analyzed by (**A**) Western blot and (**B**) qRT-PCR for YAP1 protein or mRNA respectively. (**C**) All three YAP shRNAs radiosensitized MDA-MB-231 cells (**D**) Schematic of the YAP1 regions targeted by the YAP1 short hairpins. Values shown are the means + SE of three independent experiments. In (C) p-values for comparisons between N.S.shRNA and YAP1shRNA1, YAP1shRNA2 and YAP1shRNA3 are arranged from top to bottom in each radiation dose. $^{*}p \leq 0.05$; $^{**}p \leq 0.001$ and $^{***}p \leq 0.0001$.

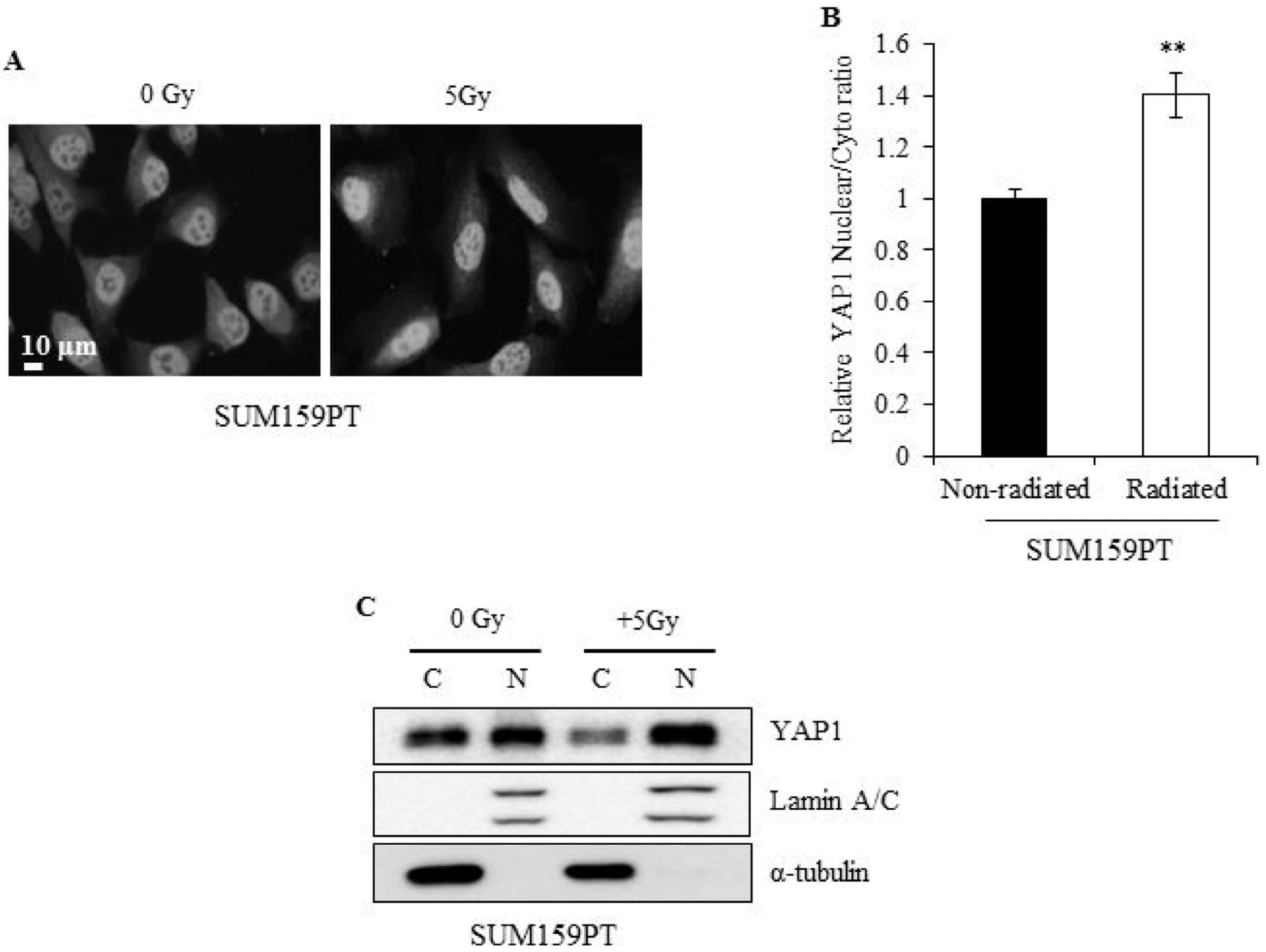

Supplementary Figure 2: Radiation induces translocation of YAP1 to the nucleus in SUM159PT cells. (**A**) Immunofluorescence analysis of YAP1 in non-irradiated and irradiated SUM159PT cells. (**B**) Ratio of nuclear and cytoplasmic YAP1 intensities quantified from images shown in (A). (**C**) Cytoplasmic (C) and nuclear (N) localization of YAP1 in non-irradiated and irradiated SUM159PT cells. Values shown are the means + SE of three independent experiments. $^{**}p \leq 0.001$

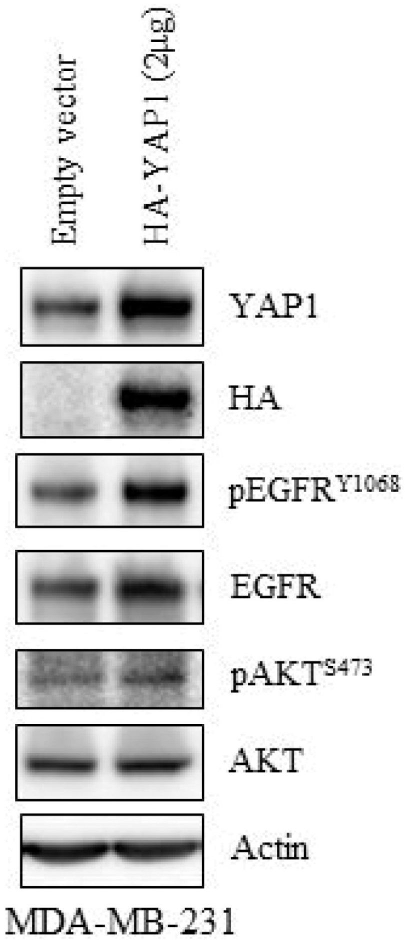

Supplementary Figure 3: YAP1 overexpression augments EGFR signaling. Lysates of MDA-MB-231 transfected with an HA-tagged YAP1 expressing vector were analyzed for HA, YAP1, pEGFRY1068, total EGFR, pAKTS473, and total AKT. Actin was used as loading control.
